# A Review on the Computational Methods for Emotional State Estimation from the Human EEG

**DOI:** 10.1155/2013/573734

**Published:** 2013-03-24

**Authors:** Min-Ki Kim, Miyoung Kim, Eunmi Oh, Sung-Phil Kim

**Affiliations:** ^1^Department of Brain and Cognitive Engineering, Korea University, Seoul 136701, Republic of Korea; ^2^Samsung Electronics, DMC R&D Center, Suwon 443742, Republic of Korea; ^3^Research and Business Foundation, Korea University, Seoul 136701, Republic of Korea

## Abstract

A growing number of affective computing researches recently developed a computer system that can recognize an emotional state of the human user to establish affective human-computer interactions. Various measures have been used to estimate emotional states, including self-report, startle response, behavioral response, autonomic measurement, and neurophysiologic measurement. Among them, inferring emotional states from electroencephalography (EEG) has received considerable attention as EEG could directly reflect emotional states with relatively low costs and simplicity. Yet, EEG-based emotional state estimation requires well-designed computational methods to extract information from complex and noisy multichannel EEG data. In this paper, we review the computational methods that have been developed to deduct EEG indices of emotion, to extract emotion-related features, or to classify EEG signals into one of many emotional states. We also propose using sequential Bayesian inference to estimate the continuous emotional state in real time. We present current challenges for building an EEG-based emotion recognition system and suggest some future directions.

## 1. Introduction

An emotional state refers to a psychological and physiological state in which emotions and behaviors are interrelated and appraised within a context [1]. From the psychological aspects, the space of the emotional state can be built from the discrete model or the dimensional model. In the discrete model, an emotional state is defined as a set of a finite number of discrete states corresponding to one of core emotions, including anger, fear, disgust, surprise, happiness, and sadness, or a combination of them [2]. The dimensional model defines an emotional state spatially with the basic dimensions of emotion such as valence and arousal and interprets an emotion through the levels of each dimension [3]. These emotion models have been used for systematical and multilateral analyses of emotion [3]. Based on the emotion models, neurophysiologic mechanisms under the emotional state have been vigorously investigated. Broadly, it has been documented that the emotional processes performed at the ventral and dorsal systems in the human brain are functionally different [4]. The ventral system, including ventral anterior cingulate gyrus and some ventral areas of prefrontal cortex (ventromedial prefrontal cortex and medial orbitofrontal cortex), is involved in the production of emotional states and the regulation of affective responses, whereas the dorsal system, including dorsal anterior cingulate gyrus, some dorsal areas of prefrontal cortex (dorsolateral, posterior dorsolateral, and mid-dorsolateral prefrontal cortex), and hippocampus, is involved in effortful emotion regulation and subsequent behavior [4, 5].

Recently, affective computing (AC) has emerged as a converging technology blending emotion into human computer interaction (HCI) [6]. AC, often called emotion aware computing, builds emotional interactions between a human and a computer by measuring the emotional state through behavioral and physiological signals and developing computational models for the emotional state [6, 7]. One of the key elements in AC is emotion recognition that estimates the emotional state of users from their behavioral and physiological responses [7]. Emotion recognition aims to advance the intelligence of computer for creating affective user interfaces and to enhance the quality of psychiatric health care.

A variety of measures have been used for emotion recognition including self-report, startle response, behavioral response, autonomic measurement, and neurophysiologic measurement [3]. Self-report readily acquires emotional responses according to the emotion modeling framework but makes it difficult to track rapid affective changes and needs to rely on the outcome from self-estimation of the emotional state [3, 8]. The startle response magnitude using electromyography (EMG) measures unconscious myoneural responses but assesses only partial aspects of emotion (e.g., arousal level) [3, 9]. Behavioral measurement detects changes in facial and/or whole-body behavior using EMG or video image, but needs an assumption that EMG signals directly correspond to a specific emotional state [3, 10]. Autonomic measurement can objectively detect emotion-related physiological responses of autonomic nervous system (ANS), such as skin conductance responses (SCRs) and heart rate variability (HRV), but only access the subspaces of the emotional state [3, 11]. Neurophysiologic measurement based on electrophysiological and neuroimaging techniques can detect a wide range of dynamics of the emotional state by directly accessing the fundamental structure in the brain from which an emotional state emerges [3, 12]. Hence, neurophysiologic measurements clearly provide the most direct and comprehensive means for emotion recognition.

A large body of research has investigated neural correlates of emotion in humans using many noninvasive sensor modalities, each presenting unique characteristics with respect to spatiotemporal resolution and mobility. Functional magnetic resonance imaging (fMRI) has been used to find cortical and subcortical structures implicated in emotional states [13]. MEG has also been used to find emotion-related neural signals from specific sources in a timely manner with fine spatial and temporal resolutions [14]. But the cost and immobility of fMRI and MEG prevents these modalities from being used for practical emotion recognition systems [15, 16]. EEG, although suffering from its poor spatial resolution and high susceptibility to noise, has been widely used to investigate the brain dynamics relative to emotion as it enables the detection of immediate responses to emotional stimuli with an excellent temporal resolution [17–21]. Being developed to become more cost-effective and mobile with increased practicability and less physical restriction [22], EEG, not without its downsides, still carries critical advantages in practical usage and therefore has been a primary option for the development of online emotion recognition systems. In fact, there have been a growing number of efforts to recognize a person's emotion in real time using EEG. For example, EmoRate developed as a commercial product (Emotiv Corp., CA, USA) detects the flow of the emotional state while user is watching a film [23]. Brown et al. proposed an EEG-based affective computer system that measures the state of valence and transmits it via a wireless link [24].

The development of an EEG-based emotion recognition system requires computational models that describe how the emotional state is represented in EEG signals and how one can estimate an emotional state from EEG signals. Despite a long history of searching for EEG indices of emotion, less attention has been paid to the computational models for emotional state estimation. Hence, we feel needs for a review of the state-of-the-art computational models for emotional state estimation to subserve the development of advanced emotion recognition methods. This paper will review the current computational methods of emotional state estimation from the human EEG with discussion on challenges and some future directions.

This paper will particularly focus on the following aspects of EEG-based emotional state estimation models. First, it will start with a quick review on EEG correlates of emotion, including definition of the emotional state space, the design of emotional stimuli, and the EEG indices of emotion. Then, it will revisit the computational methods to extract EEG features relative to emotional states and to estimate emotional states from EEG. We will also propose a mathematical approach to the estimation of continuous emotional state based on Bayesian inference.

## 2. EEG Correlates of Emotion

Finding EEG correlates of emotional states should begin with how to define the emotional state space. The emotional state space can be largely categorized into a discrete space and a continuous space. The discrete state space draws upon the discrete emotion model and contains a set of discrete experiential emotional states. The discrete emotional state comprises seven to ten core emotions such as happiness, surprise, sadness, anger, disgust, contempt, and fear [2, 25] and sometimes expands to contain a large number of emotions with the synonyms of these core emotions [25]. The continuous state space is built from the dimensional emotion model and represents an emotional state as a vector in a multidimensional space. This vector space of the continuous emotional state depends on the definition a basis. For instance, the circumplex model, developed by Russell, describes an emotional state in a two-dimensional circular space with the arousal and valence dimensions [26]. Various psychological models define emotional dimensions that subsequently constitute the basis for the emotional state space [25, 27–30].

Based on the construction of the emotional state space, the investigation of EEG correlates of emotion should also address how to determine experimental stimuli to induce emotions. Typically, emotional stimuli are selected to cover desired arousal levels and valence states, and presented in different modalities including the visual, auditory, tactile, or odor stimulation. The ground truth of the emotional state induced by a stimulus is secured by exploiting the self-ratings of subjects or using the standard stimulus sets such as the international affective picture system (IAPS) or the international affective digitized sound system (IADS). The IAPS provides a set of normative pictures for emotional stimuli to induce emotional changes and attention levels [31]. The IADS embodies acoustic stimuli to induce emotions, sometimes together with the IAPS [32]. These international affective systems are known to be independent of culture, sex, and age [33].

A number of neuropsychological studies have reported EEG correlates of emotion. These EEG features can be broadly placed in one of two domains: time domain and frequency domain. In the time domain, several components of event-related potentials (ERPs) reflected underlying emotional states [34]. These ERP components can be encapsulated in a chronological order: P1 and N1 components generated in a short latency from stimulus onset, N2 and P2 in a middle latency, and P3 and slow cortical potential (SCP) in a long latency. The ERP components of short to middle latencies have been shown to correlate with valence [34–37], whereas with the ERP components of middle to long latencies have been shown to correlate with arousal [38–41]. Basically, the computation of ERPs requires averaging EEG signals over multiple trials, rendering ERP features inappropriate for online computing. However, recent developments of the single-trial ERP computation methods increase a possibility to use ERP features for online emotional state estimation [42–46].

In the frequency domain, the spectral power in various frequency bands has been implicated in the emotional state. The alpha power varied with the valence state [47] or with discrete emotions such as happiness, sadness, and fear [18]. Specifically, the frontal asymmetry of the alpha power has been repeatedly reported as a steady correlate of valence [48]. The subsequent studies have suggested that the frontal alpha asymmetry may reflect the approach/avoidance aspects of emotion, rather than valence per se [49]. The event-related synchronization (ERS) and desynchronization (ERD) of the gamma power has been related to some emotions such as happiness and sadness [50–52]. The ERS of the theta power has also been modulated during transitions in the emotional state [18, 53–55].

Besides the waveforms and the spectral power, the interactive properties between a pair of EEG oscillations such as phase synchronization and coherence have also been implicated in emotional processes. For instance, the phase synchronization level between the frontal and right temporoparietal areas varied with the emotional states of energetic, tension, and hedonic arousal [56]. The EEG coherence across the prefrontal and posterior beta oscillations was increased by viewing high arousal images [57]. Also, increases in the gamma phase synchronization index were induced by unpleasant visual stimuli [58]. As the emotional process engages a large-scale network of the neural structures in the brain, these multichannel analyses of EEG across the brain will reveal more signatures of emotion as they do for other cognitive functions [59–64]. In short, a brief summary of the EEG correlates of emotion is presented in Table 1.

## 3. Computational Methods to Estimate Emotional States

**Figure 1:**
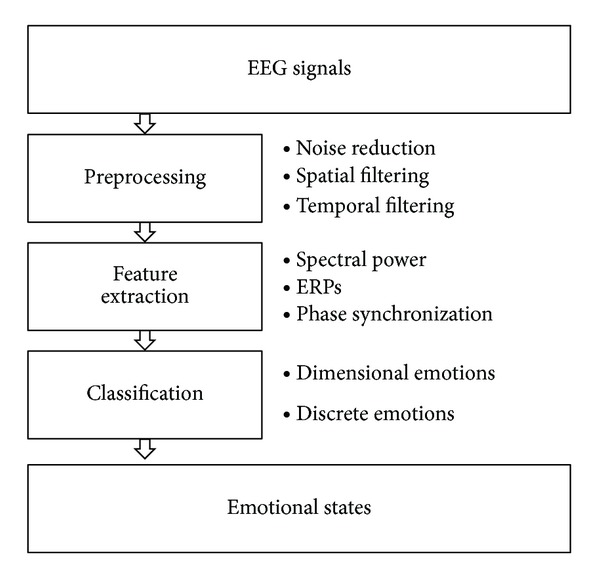
Overall emotional state estimation process. The overall emotional state estimation procedure. EEG signals are recorded during emotional situations and passed through the preprocessing step including noise reduction and spatial and temporal filtering. The features related with the emotional states such as spectral power, ERP, and phase synchronization are extracted from the preprocessed EEG signals. These features are used to estimate emotional states by classification methods.

The computational methods to estimate the emotional state have been designed based on various EEG features related to emotional processes. As most EEG analysis methods are accompanied by preprocesses for reducing the artifacts, so is the emotional state estimation method.  Figure 1 illustrates overall processing steps to estimate the emotional state from EEG signals. The recorded EEG signals in response to affective stimuli pass through the preprocessing step in which noise reduction algorithms and spatiotemporal filtering methods are employed to enhance the signal-to-noise power ratio (SNR). Then, the feature extraction step determines specific band powers, ERPs, and phase coupling indices correlated with the target emotional states. Usually, this feature selection process is optimized by mathematical methods to achieve maximum emotional estimation accuracy. The classification step estimates the most probable emotional state from the selected EEG features. The number of class depends on the definition of the emotional state space, such as the continuous state of arousal and valence, or the discrete states.

As the preprocessing methods are relatively general to a variety of EEG signal processing applications, here we focus on the feature extraction and emotion classification methods. We first review the computational methods to extract emotion-related features from EEG, followed by the classification algorithms used to estimate the emotional state from the EEG features. The feature extraction methods usually build a computational model to find emotion-related features based on neurophysiologic and neuropsychological knowledge. Unlike the feature extraction methods, the classification methods draw more upon signal processing theories such as machine learning and statistical signal processing. It has been of interest how each of these two steps impact on estimation accuracy. On one hand, the feature extraction seems to be more closely tied to estimation performance since without pointing to the very features correlated with emotion, it is implausible to build a correct model. On the other hand, the classification algorithms should also be carefully designed to fit to the characteristics of the feature space; for instance, using a linear classifier for highly nonlinear feature structures would not make much sense. In general, one should weigh coherence between a feature space and a classifier for increasing estimation accuracy.

### 3.1. Feature Extraction Methods

As for valence-related features, it has been shown that positive and negative emotions induce asymmetric modulations in the frontal alpha power of EEG, leading to a relative decrease in the left frontal alpha power for positive emotions and a decrease in the right for negative emotions [65]. This frontal alpha asymmetry provides an effective index for valence by computing a difference between the left and right alpha powers, here denoted as *L* and *R* respectively, divided by the sum of both:

(1) $$\begin{array}{c} \text{Index}=\frac{L-R}{L+R}. \end{array}$$

The computation of the spectral power in the alpha band has been executed by a number of methods, including the squares of the EEG amplitude filtered through an alpha bandpass filter [53], Fourier transform [66], power spectral density [18, 21], and wavelet transform [7, 67, 68]. Most of these methods are well established and can readily be implemented in real time.

As for arousal-related features, one can extract the spectral power features such as the frontal midline theta power similar to the alpha power. Recently, more advanced computational methods have been proposed to evaluate emotional arousal. For instance, Asymmetry index (AsI) assesses emotion elicitation by computing a multidimensional directed information (MDI) between EEG channels [69]:

(2) $$\begin{array}{c} S_{n}=S_{n}^{XY}+S_{n}^{YX},\\ S_{f}=S_{f}^{XY}+S_{f}^{YX},\\ \text{AsI}=\left(S_{n}-S_{f}\right)\times \frac{\sqrt{2}}{2}. \end{array}$$

*S*_*f*_^*XY*^ indicates the total amount of information flowing from left hemisphere signals, *X*, to right hemisphere signals, *Y*, when the subject has emotional feelings. *S*_*f*_ refers to the total bidirectional information with emotion. *S*_*n*_^*XY*^ indicates the same directional information from *X* to *Y* but when the subject does not have emotional feelings, and *S*_*n*_ refers to the total bidirectional information without emotion. AsI can effectively indicate whether an emotional state is elicited or not [69]. Besides AsI, the variance of potentials from a specific channel over different EEG channels has been used as an emotion-related feature [68]. Also, the entropy of EEG signals has been used to extract information related to emotion from intrusive noise [68].

As for individual discrete emotions, a typical approach is to search through all the possible EEG channels, spectral bands, and time segments for a set of features that maximizes the accuracy of emotional state estimation. This approach adopts a greedy search method with supervised learning, often resulting in different optimal feature sets for each individual. To overcome this issue of subject-by-subject variability, a higher order crossing (HOC) analysis was developed to implement a user-independent emotion recognition system [70]. The HOC analysis aims to find EEG features with respect to six affective traits, including surprise, disgust, anger, fear, happiness, and sadness [70]. The HOC model is given as:

(3) Dn=∑i=2m(Xi[n]−Xi−1[n])2,i  =  1,…,m; n  =  1,2,3,…,FVHOC=[D1,D2,…,DL], 1<L≤Maximum  order.

*D*_*n*_ is the simplified version of the HOC feature that counts the number of zero-crossing from a high-pass filtered, standardized EEG time series. Zero-crossing indicates an event at which the signal amplitude passed through a zero-line with the change of polarity. The zero-crossing counts often represent oscillation properties more robustly than the spectral power. A vector of the simple HOCs is constructed to contain the features related to emotion. A higher value of *n* means decreases in the discrimination power of the simple HOC because different processes can yield almost the same *D*_*n*_.  *X*_*i*_[*n*] indicates a binary time series with zeros and ones: at time instant *i* where *X*_*i*_[*n*] = 0 if the amplitude of the filtered signal is negative and *X*_*i*_[*n*] = 1 otherwise. *m* indicates the length of the time series *X*_*i*_. The EEG feature vector is defined as *FV*_HOC_ that consists of multiple simple HOCs [70]. The computational methods to extract emotional features from EEG are summarized in Table 2.

### 3.2. Emotion Classification Methods

The EEG feature vector provides observations from which an emotional state can be inferred. Commonly, a classifier has been used for decoding the feature vector into one of possible emotional states. A number of classification methods have been used for emotional state estimation, including discriminant analysis (DA), support vector machine (SVM), k-nearest neighbor (k-NN), and the Mahalanobis distance (MD) based method. DA performs dimensionality reduction in a high-dimensional feature space onto a low-dimensional space with an aim to maximize the Fisher discriminant ratio, *S*, of between-class scatter, *C*_*B*_, to within-class scatter, *C*_*W*_, [42, 71–76].

(4) $$\begin{array}{c} S=\mathrm{tr}\left(C_{B}C_{W}^{-1}\right). \end{array}$$

A larger *S* value indicates greater separation between classes. The dimensionality of the low-dimensional space varies from one up to the number of classes minus one.

SVM is derived from DA but determines a decision boundary in a kernel space instead of the original feature space. SVM finds an optimal hyperplane, *H*(*x*), and the hypermargin of the decision boundary in the feature space using a supervised learning method. The classifier *u*(*x*_*i*_) classifies a new input feature vector *x*_*j*_ using a classification rule given by

(5) $$\begin{array}{c} u\left(x_{i}\right)=\mathrm{sgn}\left(\sum_{x_{i}\in v}\alpha_{i}t_{i}F\left(x_{i},x_{j}\right)+b\right),\;t_{i}\in \text{target}\;\text{space}. \end{array}$$

Here, **v** indicates a set of the support vectors that are used to determine the maximum margin hyperplane, and *F*(*x*_*i*_, *x*_*j*_) denotes the kernel function of the SVM classifier. *b* denotes an offset parameter, *x*_*i*_ does training input vectors, and *α*_*i*_ does nonzero weights on each support vector [7, 77–80]. Various kernel functions have been proposed such as the Gaussian function or polynomials. SVM offers advantages of good generalizability for nonlinear feature spaces.

The k-NN algorithm determines the class of a new feature vector according to the number of nearest vectors in the training set surrounding a new feature vector [73, 81]. k is a parameter determining the encircled boundary. The k-NN algorithm depends on how to define a distance between feature vectors, which is subject to be affected by the curse of dimensionality [81, 82].

The MD-based method, has been widely used in the clustering analysis, not only for distance, but also with correlation coefficient and the standard deviation [83, 84]:

(6) $$\begin{array}{c} \text{MD}={\left(x-\mu_{c}\right)}^{T}C_{c}^{-1}\left(x-\mu_{c}\right). \end{array}$$

**C** ^−1^ and ***μ***_*c*_ indicate the inverse of the covariance matrix and the mean vector of a class *c*, respectively. MD converges to Euclidean distance when the covariance matrix of feature vectors becomes the identify matrix [84]. Basically, when a new feature vector arrives, the MD-based classifier compares the distance of the vector to each class using MD and chooses the class with the smallest distance. The classification methods that have been used for emotional state estimation are summarized in Table 2.

## 4. A Generative Model for Online Tracking of Emotional States

As described earlier, most computational models estimating emotional states have focused on the discrete state space and classified EEG features into one of a finite number of emotional states. This approach generally suits well to the case of a static determination of which emotion is induced by a given stimulus. Yet, for the development of an online emotion recognition system, where continuous tracking of the emotional state may play an important role, the current approach might be suboptimal because they do not take temporal dynamics of the emotional state into account. Another downside of the current approach originates from their direct modeling framework. A model in this framework builds a direct input-output mapping from the observed EEG signal to the emotional state. Although this framework may be able to provide a reasonable solution just for the purpose of improving classification accuracy, it does not exploit prior information of the emotional state as well as dynamics of the emotional state. These shortcomings make it difficult to gain useful insights on the neural mechanism of emotion. Also, it is often desirable to incorporate prior information of the dynamics of the emotional state within a model, especially for tracking emotional state continuously over time.

To address these issues, we propose a computational modeling approach based on the generative modeling framework [85–87]. Our approach focuses on tracking the change of the emotional state over time from EEG signal. In this approach, a generative model depicts how EEG signal is generated from a hidden emotional state. Also, a prior model explains how the emotional state changes over time. Integrating these two models, we infer a most likely emotional state from an observed EEG signal. Differences between the generative and direct models can be illustrated in a probabilistic view where a goal is to estimate a conditional probability of emotional state variables given EEG observations as accurately as possible. Suppose that a random vector **x** denotes hidden emotional states and a random vector **y** denotes observed EEG data (e.g., an EEG feature vector). An estimation model aims to optimize a parameter set, *θ*, for the following conditional probability:

(7) $$\begin{array}{c} p\left(x\;|\;y,\theta \right). \end{array}$$

A direct model forms a functional relationship from **y** to **x** with *θ*, the parameter set of a function *f*(·),

(8) $$\begin{array}{c} x=f\left(y;\theta \right)+e, \end{array}$$

where **e** is a residual vector. In many cases, the residual vector is assumed to follow the Gaussian distribution. Parameter estimation of *θ* can be accomplished by many standard solutions such as maximum likelihood [88]. On the other hand, a generative model uses maximum a posteriori (MAP) or the Bayesian inference to estimate the conditional probability,

(9) $$\begin{array}{c} p\left(x\;|\;y,\theta \right)=\frac{1}{D}p\left(y\;|\;x,\theta \right)p\left(x\right), \end{array}$$

where *D* represents a constant representing the integral of *p*(**y**). The posterior *p*(**x** | **y**,  *θ*) is estimated by the product of *p*(**y** | **x**,  *θ*), the likelihood of observation **y** given a state **x**, and *p*(**x**), the prior of the state **x**. The parameter set *θ* is used to model a generative relation from **x** to **y**. In terms of the EEG correlate of the emotional state, the likelihood describes how the observed EEG signal is generated from an emotional state, the prior describes a probability of each emotional state, and the posterior describes which emotional state most likely elicits the observed EEG signal.

Here, we extend this generative approach to take into account the temporal dynamics of the emotional state. We use sequential Bayesian inference to track a time-varying emotional state from EEG signal [89]. To this end, we first assume that the emotional state is defined in a continuous space. An example of a continuous state space consists of two emotional dimensions, such as valence and arousal. The valence dimension ranges from negative values to positive values. The arousal dimension ranges from low to high arousal levels. A key point is that an emotional state varies over a continuous space, instead of altering between discrete values. This does not mean that we need to assign an explicit emotion to every possible point in the emotional state space. A specific area or volume in the state space can represent a single emotion.

The generative model is then formulated as follows. Let **x**_*t*_ be an emotional state vector and **y**_*t*_ an EEG signal vector at time instant *t*. **x**_*t*_ contains a set of emotional state variables (e.g., **x**_*t*_ = [*x*_1,*t*_, *x*_2,*t*_, *x*_3,*t*_], where *x*_1,*t*_ is the valence dimension, *x*_2,*t*_ is the arousal dimension, and *x*_3,*t*_ is the dominance dimension). **y**_*t*_ contains a set of EEG features selected to be related to emotion (e.g., the power of certain frequency band at a selected channel). The goal of the model is to find the most probable emotional state given a series of observation from the beginning, **y**_1_,…, **y**_*t*_ (assuming observation begins at *t* = 1). The posterior is formed as

(10) $$\begin{array}{c} p\left(x_{t}\;|\;y_{1},\ldots y_{t}\right). \end{array}$$

The posterior can be rewritten as a recursive equation,

(11) p(xt ∣ y1,…,yt)  =p(yt ∣ xt)∫p(xt ∣ xt−1)p(xt−1 ∣ yt,…,yt−1)dxt−1.

Note that the likelihood, *p*(**y**_*t*_ | **x**_*t*_), depends only on the current time *t*. The prior, *p*(**x**_*t*_ | **y**_*t*−1_), represents state transition from *t* − 1 to *t*, assuming the first-order Markov process. The dynamics of emotional state is embedded in the prior, whereas the generative process of the EEG features from an emotional state is modeled by the likelihood. The integral can be approximately computed by a number of methods with different model assumptions [89].

As this approximation relies on the recursion of the posterior, inference of an emotional state from EEG signal operates sequentially over time. This property enables our model fit well to the purpose of tracking emotional states continuously. In fact, the sequential Bayesian inference model (or called a Bayesian filter) has been widely adopted for many neuroengineering studies (e.g., see [90–94]). Our model may provide an effective way for online emotion aware computing, especially when we need to keep track of changes in the emotional state from EEG measurements continuously over time, for instance, tracking emotional changes while a subject is watching movies [95].

## 5. Discussion

In this paper, we overviewed the computational methods used for emotional state estimation. We first briefly gave an overview of the EEG correlates of emotion. Then, we revisited the computational methods to extract EEG features correlated with the continuous and discrete emotional states. We also described the classification methods to discriminate a particular emotional state from EEG features. Finally, we proposed a computational approach based on the generative modeling framework that may suit well to tracking the emotional state over time. These computational methods for emotional state estimation will serve as a key element for practical online emotion recognition systems for affective computing.

While affective computing has attracted attentions in the HCI field with a promise to develop a novel user interface, the development of the computational methods to estimate the emotional state still requires further understanding of emotion processes and their neurophysiologic substrates [96]. Especially, the estimation of emotional states from the human EEG has been posed only as a relatively simple classification problem with a few discrete emotions. The development of a real-time emotional state tracking system would require a more rigorous definition of the emotional state space suitable for estimation models.

Exploration of the EEG signatures of emotion that can span a broad area of the emotional state space or represent a number of different discrete emotions should continue. Such investigations may need to overcome many existing challenges. In particular, finding such EEG signatures of emotion that are invariant across individuals will be important for general emotion recognition systems [69]. As the emotion-related features have been mostly found in the frontal EEG signal, online algorithms to overcome the eye movement artifact should be continuously developed [97–99]. Also, bringing the EEG-based emotion recognition system out to the normal users would require a simple yet efficient EEG sensor. A new EEG sensor should meet some criteria such as stabilization of a signal to noise ratio (SNR), reduction of noise elicited from hair, optimization of active dry electrodes, development of multi-channel wireless communication, and sustainment of the quality of EEG signals over a long period [100–103]. Many previous studies have estimated the emotional state by analyzing the EEG responding to specific emotional stimuli. However, this emotion-induction paradigm has a limitation that the EEG signals can be modulated by the stimulus properties irrelevant to emotion [21]. Hence, a computational model that can predict the emotional state with various stimuli may be required for real-world applications.

The computational methods to estimate the emotional state may improve further with several advances in computational models. First, a model that can associate the dynamics of EEG signal with the dynamics of cognitive emotional process will provide a basis for constructing a novel emotional state estimation method. The current methods only capture the static properties in the EEG pattern in response to emotional stimuli. If a new model can embrace the temporal dynamics of emotional information processing in the human cognitive system and find EEG correlates of those dynamical properties, it will estimate the emotional state more precisely. Second, the quest for novel EEG signatures of the emotional state should be pursued. In particular, interactive properties between EEG signals such as cross-frequency coupling and effective connectivity pattern may be worth exploring to find novel EEG correlates of emotion. Third, inference of emotion-related information from high-dimensional and nonlinear EEG data poses an interesting problem to develop and apply the state-of-the-art machine learning algorithms. So far, only a few basic learning algorithms have been applied for emotional state estimation, but it is likely that emotion recognition would benefit from more advanced statistical learning and pattern recognition algorithms. With these advances, we foresee that the computational models of emotional estimation would play a key role in future consumer devices. Before long, they can bring serendipity to device users by estimating emotional states in a natural and nonintrusive way.

## Figures and Tables

**Table 1:** EEG correlates of emotion.

Authors	Year	#subjects	Stimulus (duration)	#EEG channels	Channel location	Emotional state	EEG features	Effects
Davidson et al. [48]	1990	37	Emotional film clips (60 sec)	8	F3, F4, C3, C4, T3, T4, P3, and P4	Happiness, disgust	Alpha power	Left-frontal: happiness < disgust Right-frontal: happiness > disgust Left-anterior temporal: happiness < disgust Right-anterior temporal: happiness > disgust

Aftanas et al. [54]	2001	22	IAPS (7 sec)	128	IS	Valence (+/−)	Theta power (ERD/ERS)	Anterior temporal region (left-hemisphere) Negative: left < right Positive: left > right Parietotemporal region Negative: left < right Positive: left < right

Keil et al. [51]	2001	10	IAPS (1 sec)	128	IS	Arousal	Gamma power	Gamma power (46–65 Hz, 500 ms): arousing ↑

Kemp et al. [104]	2002	16	IAPS (13 Hz)	64	IS	Valence (+/−)	SSVEP amplitude	Negative: SSVEP ↓ at the bilateral anterior frontal area

Pollatos et al. [105]	2005	44	IAPS (6 sec)	61	IS	Arousal	ERP/ECG	Good heartbeat perceivers show higher P300 peak

Balconi and Lucchiari [18]	2006	20	Ekman's picture set (500 ms)	14	Fz, Cz, Pz, Oz, F3, F4, C3, C4, T3, T4, P3, P4, O1, and O2	Neutral versus emotions (happy, sad, angry, and, fearful)	ERD (alpha, beta, delta, and theta)	ERD% of theta (150–250 ms) at the anterior regions Emotional state > neutral state

Baumgartner et al. [106]	2006	24	IAPS + music (16 pictures/4.375 s)	16	F7, F3, FT7, FC3, F4, F8, FC4, FT8, TP7, CP3, P7, P3, CP4, TP8, P4, and P8	Happiness, sadness, and fear	Alpha power	Combining music with pictures evokes more intensive emotional experience

Sammler et al. [55]	2007	18	Music (22–44 sec)	63	IS	Valence (+/−)	Theta power	Frontal midline theta power is increased by positive emotion

Balconi and Mazza [47]	2009	19	Ekman's picture set (30 ms, 200 ms)	32	IS	Anger, fear, surprise, disgust, and happiness	Alpha power (ERD)	Right-frontal activity increase for negative emotions

Li and Lu [107]	2009	10	Picture (6 sec)	62	IS	Happiness, sadness	Gamma ERD	Gamma ERD for emotional stimuli

Petrantonakis and Hadjileontiadis [70]	2010	16	Ekman's picture set	3	Fp1, Fp2, and F3/F4 (bipolar)	Happiness, sadness, surprise, anger, fear, and disgust	Alpha and beta power	Higher-order crossing index improves performance

Lithari et al. [108]	2010	28	IAPS (2 sec)	19	IS	Arousal	ERP (N100, N200)	ERP peaks increase for unpleasant stimuli in female

Petrantonakis [69]	2011	16	IAPS (5 sec)	8	F3, F4, C3, C4, T3, T4, P3, P4	Arousal/valence	Alpha and beta power	Asymmetry index can detect arousal levels

Park et al. [109]	2011	34	Video: comic, horror, sadness, and peaceful (10 min)	32	IS	Fear, sadness, peace, and happiness	Alpha, beta, and gamma	Fear emotion: beta wave ↑ at the left temporal lobe happy emotion: alpha wave ↓ at the C4 peaceful state: gamma wave ↑ at the T5, alpha wave ↓ at the CP5 Sadness emotion: alpha wave ↓ at the left temporal lobe

Degabriele et al. [110]	2011	18	Emotional go/no-go inhibition task (300 ms)	24	IS	Happiness, sadness	ERP (P100 and N170)	P100 amplitude: happiness > sadness N170 amplitude: bipolar disorder is less at all emotional state than normal subjects

**Table 2:** Emotional state estimation model.

Author	Year	Stimulus (duration)	#channels	Channel location	Emotional states	EEG features	Feature extraction methods	Classifier	Accuracy (%)/#classes	Online versus offline
Li and Lu [107]	2009	Picture (6 sec)	62	IS	Happiness, sadness	Gamma ERD	CSP	SVM	93.5/2	Offline

Murugappan et al. [81]	2010	Video (−)	62	IS	Happiness, surprise, fear, disgust, and neutral	Delta, theta, alpha, beta, and gamma	Wavelet transform	kNN, LDA	83.04/5 (62 channels) 79.17/5 (24 channels)	Offline

Lin et al. [111]	2010	Music (30 sec)	24	Fp1-Fp2, F7-F8, F3-F4, FT7-FT8, FC3-FC4, T7-T8, P7-P8, C3-C4, TP7-TP8, CP3-CP4, P3-P4, O1-O2 (bipolar)	Joy, anger, sadness, and pleasure	Delta, theta, alpha, beta, and gamma	Short-time Fourier transform	SVM	82.29/4	Offline

Petrantonakis and Hadjileontiadis [70]	2010	Ekman's picture set (−)	3	Fp1, Fp2, F3/F4 (bipolar)	Happiness, sadness, surprise, anger, fear, and disgust	ERD/ERS	Higher-order crossing	QDA SVM	62.3/6 (QDA) 83.33/6 (SVM)	Offline

Hosseini et al. [112]	2010	IAPS (3 sec)	5	FP1, FP2, T3, T4, Pz	Calm-neutral, negative-excited	Delta, theta, alpha, beta, and gamma	Wavelet coefficients, Higuchi's algorithm	Elman ANN	82.7/2	Offline

Wang et al. [113]	2011	Video (4 min)	62	IS	Joy, relax, sad, and fear	Alpha, beta, and gamma	Minimum redundancy maximum relevance method	SVM	66.5/4	Offline

Brown et al. [24]	2011	IAPS (6 sec)	8	Fp1, Fp2, F3, F4, F7, F8, C3, C4	Positive, negative, and neutral	Alpha1 (6–8 Hz), Alpha2 (8–10 Hz), Alpha3 (10–12 Hz)	Spectral power features	kNN	85.0/3	Online

Petrantonakis [69]	2011	IAPS (5 sec)	8	F3, F4, C3, C4, T3, T4, P3, P4	Arousal, valence	Alpha and beta power	Asymmetry index	SVM	94.4/2	Offline

Stelios and Hadjidimitriou [114]	2012	Music (60 sec)	14	AF3, F7, F3, FC5, T7, P7, O1, O2, P8, T8, FC6, F4, F8, AF4	Arousal, neutral	Beta and gamma	Time-frequency (TF) analysis	SVM KNN QDA MDA	86.52/2	Offline

Konstantinidis et al. [22]	2012	IAPS (2D emotional space)	19	IS	Arousal, valence	ERP (N100, N200)	Amplitude of ERP	SVM	81.3/4	Online

(i) SVM: support vector machine; kNN: k-nearest neighbor; LDA: linear discriminant analysis; QDA: quadratic discriminant analysis; MDA: Mahalanobis distance based discriminant analysis; SOM: self-organization map; ANN: artificial neural networks.

(ii) IS: channel configuration followed the 10/20 International System.

## References

[1] Scherer KR (2005). What are emotions? and how can they be measured?. *Social Science Information*.

[2] Barrett LF (1998). Discrete emotions or dimensions? the role of valence focus and arousal focus. *Cognition and Emotion*.

[3] Mauss IB, Robinson MD (2009). Measures of emotion: a review. *Cognition and Emotion*.

[4] Phillips ML, Drevets WC, Rauch SL, Lane R (2003). Neurobiology of emotion perception II: implications for major psychiatric disorders. *Biological Psychiatry*.

[5] Phillips ML, Drevets WC, Rauch SL, Lane R (2003). Neurobiology of emotion perception I: the neural basis of normal emotion perception. *Biological Psychiatry*.

[6] Luneski A, Konstantinidis E, Bamidis PD (2010). Affective medicine: a review of affective computing efforts in medical informatics. *Methods of Information in Medicine*.

[7] Frantzidis CA, Bratsas C, Papadelis CL, Konstantinidis E, Pappas C, Bamidis PD (2010). Toward emotion aware computing: an integrated approach using multichannel neurophysiological recordings and affective visual stimuli. *IEEE Transactions on Information Technology in Biomedicine*.

[8] Robinson MD, Clore GL (2002). Belief and feeling: evidence for an accessibility model of emotional self-report. *Psychological Bulletin*.

[9] Cuthbert BN, Bradley MM, Lang PJ (1996). Fear and anxiety: theoretical distinction and clinical test. *Psychophysiology*.

[10] Adolphs R (2002). Neural systems for recognizing emotion. *Current Opinion in Neurobiology*.

[11] Christie IC, Friedman BH (2004). Autonomic specificity of discrete emotion and dimensions of affective space: a multivariate approach. *International Journal of Psychophysiology*.

[12] Panksepp J (2007). Neuro-psychoanalysis may enliven the mindbrain sciences. *Cortex*.

[13] Vytal K, Hamann S (2010). Neuroimaging support for discrete neural correlates of basic emotions: a voxel-based meta-analysis. *Journal of Cognitive Neuroscience*.

[14] Peyk P, Schupp HT, Elbert T, Junghöfer M (2008). Emotion processing in the visual brain: a MEG analysis. *Brain Topography*.

[15] Hämäläinen M, Hari R, Ilmoniemi RJ, Knuutila J, Lounasmaa OV (1993). Magnetoencephalography—theory, instrumentation, and applications to noninvasive studies of the working human brain. *Reviews of Modern Physics*.

[16] Ray A, Bowyer SM (2010). Clinical applications of magnetoencephalography in epilepsy. *Annals of Indian Academy of Neurology*.

[17] Davidson PR, Jones RD, Peiris MT (2007). EEG-based lapse detection with high temporal resolution. *IEEE Transactions on Biomedical Engineering*.

[18] Balconi M, Lucchiari C (2006). EEG correlates (event-related desynchronization) of emotional face elaboration: a temporal analysis. *Neuroscience Letters*.

[19] Balconi M, Pozzoli U (2008). Event-related oscillations (ERO) and event-related potentials (ERP) in emotional face recognition. *International Journal of Neuroscience*.

[20] Beaton AA, Fouquet NC, Maycock NC, Platt E, Payne LS, Derrett A (2012). Processing emotion from the eyes: a divided visual field and ERP study. *Laterality*.

[21] Bekkedal MY, Rossi J, Panksepp J (2011). Human brain EEG indices of emotions: delineating responses to affective vocalizations by measuring frontal theta event-related synchronization. *Neuroscience & Biobehavioral Reviews*.

[22] Konstantinidis EI, Frantzidis CA, Pappas C, Bamidis PD (2012). Real time emotion aware applications: a case study employing emotion evocative pictures and neuro-physiological sensing enhanced by Graphic Processor Units. *Computer Methods and Programs in Biomedicine*.

[23] Sourina O, Liu Y A fractal-based algorithm of emotion recognition from EEG using arousal-valence model.

[24] Brown L, Grundlehner B, Penders J (2011). Towards wireless emotional valence detection from EEG. *IEEE Engineering in Medicine and Biology Society*.

[25] Russell JA (2003). Core affect and the psychological construction of emotion. *Psychological Review*.

[26] Schaefer ES (1959). A circumplex model for maternal behavior. *Journal of Abnormal and Social Psychology*.

[27] Mehrabian A (1980). *Basic Dimensions for a General Psychological Theory :Implications for Personality, Social, Environmental, and Developmental Studies*.

[28] Fontaine JR, Scherer KR, Roesch EB, Ellsworth PC (2007). The world of emotions is not two-dimensional. *Psychological Science*.

[29] Hamann S (2012). Mapping discrete and dimensional emotions onto the brain: controversies and consensus. *Trends in Cognitive Sciences*.

[30] Rubin DC, Talarico JM (2009). A comparison of dimensional models of emotion: evidence from emotions, prototypical events, autobiographical memories, and words. *Memory*.

[31] Mikels JA, Fredrickson BL, Larkin GR, Lindberg CM, Maglio SJ, Reuter-Lorenz PA (2005). Emotional category data on images from the international affective picture system. *Behavior Research Methods*.

[32] Redondo J, Fraga I, Padrón I, Piñeiro A (2008). Affective ratings of sound stimuli. *Behavior Research Methods*.

[33] Ribeiro RL, Pompéia S, Amodeo Bueno OF (2005). Comparison of Brazilian and American norms for the International Affective Picture System (IAPS). *Revista Brasileira de Psiquiatria*.

[34] Olofsson JK, Nordin S, Sequeira H, Polich J (2008). Affective picture processing: an integrative review of ERP findings. *Biological Psychology*.

[35] Codispoti M, Ferrari V, Bradley MM (2007). Repetition and event-related potentials: distinguishing early and late processes in affective picture perception. *Journal of Cognitive Neuroscience*.

[36] Olofsson JK, Polich J (2007). Affective visual event-related potentials: arousal, repetition, and time-on-task. *Biological Psychology*.

[37] Gianotti LRR, Faber PL, Schuler M, Pascual-Marqui RD, Kochi K, Lehmann D (2008). First valence, then arousal: the temporal dynamics of brain electric activity evoked by emotional stimuli. *Brain Topography*.

[38] Bernat E, Bunce S, Shevrin H (2001). Event-related brain potentials differentiate positive and negative mood adjectives during both supraliminal and subliminal visual processing. *International Journal of Psychophysiology*.

[39] Cuthbert BN, Schupp HT, Bradley MM, Birbaumer N, Lang PJ (2000). Brain potentials in affective picture processing: covariation with autonomic arousal and affective report. *Biological Psychology*.

[40] Roschmann R, Wittling W (1992). Topographic brain mapping of emotion-related hemisphere asymmetries. *International Journal of Neuroscience*.

[41] Yee CM, Miller GA (1987). Affective valence and information processing. *Electroencephalography and Clinical Neurophysiology*.

[42] Blankertz B, Lemm S, Treder M, Haufe S, Müller KR (2011). Single-trial analysis and classification of ERP components—a tutorial. *NeuroImage*.

[43] Hu L, Liang M, Mouraux A, Wise RG, Hu Y, Iannetti GD (2011). Taking into account latency, amplitude, and morphology: improved estimation of single-trial ERPs by wavelet filtering and multiple linear regression. *Journal of Neurophysiology*.

[44] Ahmadi M, Quian Quiroga R (2012). Automatic denoising of single-trial evoked potentials. *NeuroImage*.

[45] Vanderperren K, Mijovic B, Novitskiy N (2013). Single trial ERP reading based on parallel factor analysis. *Psychophysiology*.

[46] Jarchi D, Sanei S, Principe JC, Makkiabadi B (2011). A new spatiotemporal filtering method for single-trial estimation of correlated ERP subcomponents. *IEEE Transactions on Biomedical Engineering*.

[47] Balconi M, Mazza G (2009). Brain oscillations and BIS/BAS (behavioral inhibition/activation system) effects on processing masked emotional cues. ERS/ERD and coherence measures of alpha band. *International Journal of Psychophysiology*.

[48] Davidson RJ (1992). Anterior cerebral asymmetry and the nature of emotion. *Brain and Cognition*.

[49] Gotlib IH, Ranganath C, Rosenfeld JP (1998). Frontal EEG alpha asymmetry, depression, and cognitive functioning. *Cognition and Emotion*.

[50] Balconi M, Lucchiari C (2008). Consciousness and arousal effects on emotional face processing as revealed by brain oscillations. A gamma band analysis. *International Journal of Psychophysiology*.

[51] Keil A, Müller MM, Gruber T, Wienbruch C, Stolarova M, Elbert T (2001). Effects of emotional arousal in the cerebral hemispheres: a study of oscillatory brain activity and event-related potentials. *Clinical Neurophysiology*.

[52] Müller MM, Keil A, Gruber T, Elbert T (1999). Processing of affective pictures modulates right-hemispheric gamma band EEG activity. *Clinical Neurophysiology*.

[53] Aftanas LI, Reva NV, Varlamov AA, Pavlov SV, Makhnev VP (2004). Analysis of evoked EEG synchronization and desynchronization in conditions of emotional activation in humans: temporal and topographic characteristics. *Neuroscience and Behavioral Physiology*.

[54] Aftanas LI, Varlamov AA, Pavlov SV, Makhnev VP, Reva NV (2001). Affective picture processing: event-related synchronization within individually defined human theta band is modulated by valence dimension. *Neuroscience Letters*.

[55] Sammler D, Grigutsch M, Fritz T, Koelsch S (2007). Music and emotion: electrophysiological correlates of the processing of pleasant and unpleasant music. *Psychophysiology*.

[56] Wyczesany M, Grzybowski SJ, Barry RJ, Kaiser J, Coenen AML, Potoczek A (2011). Covariation of EEG synchronization and emotional state as modified by anxiolytics. *Journal of Clinical Neurophysiology*.

[57] Miskovic V, Schmidt LA (2010). Cross-regional cortical synchronization during affective image viewing. *Brain Research*.

[58] Martini N, Menicucci D, Sebastiani L (2012). The dynamics of EEG gamma responses to unpleasant visual stimuli: from local activity to functional connectivity. *NeuroImage*.

[59] Fell J, Axmacher N (2011). The role of phase synchronization in memory processes. *Nature Reviews Neuroscience*.

[60] Sauseng P, Klimesch W (2008). What does phase information of oscillatory brain activity tell us about cognitive processes?. *Neuroscience and Biobehavioral Reviews*.

[61] Fries P (2009). Neuronal gamma-band synchronization as a fundamental process in cortical computation. *Annual Review of Neuroscience*.

[62] Pockett S, Holmes MD (2009). Intracranial EEG power spectra and phase synchrony during consciousness and unconsciousness. *Consciousness and Cognition*.

[63] Melloni L, Molina C, Pena M, Torres D, Singer W, Rodriguez E (2007). Synchronization of neural activity across cortical areas correlates with conscious perception. *Journal of Neuroscience*.

[64] Doesburg SM, Roggeveen AB, Kitajo K, Ward LM (2008). Large-scale gamma-band phase synchronization and selective attention. *Cerebral Cortex*.

[65] Tomarken AJ, Davidson RJ, Henriques JB (1990). Resting frontal brain asymmetry predicts affective responses to films. *Journal of Personality and Social Psychology*.

[66] Güntekin B, Basar E (2007). Emotional face expressions are differentiated with brain oscillations. *International Journal of Psychophysiology*.

[67] Gross E, El-Baz AS, Sokhadze GE, Sears L, Casanova MF, Sokhadze EM (2012). Induced eeg gamma oscillation alignment improves differentiation between autism and adhd group responses in a facial categorization task. *Journal of Neurotherapy*.

[68] Murugappan M, Nagarajan R, Yaacob S (2011). Combining spatial filtering and wavelet transform for classifying human emotions using EEG Signals. *Journal of Medical and Biological Engineering*.

[69] Petrantonakis LJHPanagiotisC (2011). A novel emotion elicitation index using frontal brain asymmetry for enhanced EEG-based emotion recognition. *IEEE Transactions on Information Technology in Biomedicine*.

[70] Petrantonakis PC, Hadjileontiadis LJ (2010). Emotion recognition from EEG using higher order crossings. *IEEE Transactions on Information Technology in Biomedicine*.

[71] Bandt C, Weymar M, Samaga D, Hamm AO (2009). A simple classification tool for single-trial analysis of ERP components. *Psychophysiology*.

[72] Horst RL, Donchin E (1980). Beyond averaging. II. Single-trial classification of exogenous event-related potentials using stepwise discriminant analysis. *Electroencephalography and Clinical Neurophysiology*.

[73] Kolodyazhniy V, Kreibig SD, Gross JJ, Roth WT, Wilhelm FH (2011). An affective computing approach to physiological emotion specificity: toward subject-independent and stimulus-independent classification of film-induced emotions. *Psychophysiology*.

[74] Poolman P, Frank RM, Luu P, Pederson SM, Tucker DM (2008). A single-trial analytic framework for EEG analysis and its application to target detection and classification. *NeuroImage*.

[75] Wang C, Xiong S, Hu X, Yao L, Zhang J (2012). Combining features from ERP components in single-trial EEG for discriminating four-category visual objects. *Journal of Neural Engineering*.

[76] Zhang Y, Zhao Q, Jin J, Wang X, Cichocki A (2012). A novel BCI based on ERP components sensitive to configural processing of human faces. *Journal of Neural Engineering*.

[77] Guler I, Ubeyli ED (2007). Multiclass support vector machines for EEG-signals classification. *IEEE Transactions on Information Technology in Biomedicine*.

[78] Gouizi K, Bereksi Reguig F, Maaoui C (2011). Emotion recognition from physiological signals. *Journal of Medical Engineering & Technology*.

[79] Martisius I, Damasevicius R, Jusas V, Birvinskas D (2012). Using higher order nonlinear operators for SVM classification of EEG data. *Elektronika ir Elektrotechnika*.

[80] Guo L, Wu Y, Zhao L, Cao T, Yan W, Shen X (2011). Classification of mental task from EEG signals using immune feature weighted support vector machines. *IEEE Transactions on Magnetics*.

[81] Murugappan M, Nagarajan R, Yaacob S (2010). Combining spatial filtering and wavelet transform for classifying human emotions using EEG Signals. *Journal of Medical and Biological Engineering*.

[82] Rafiul Hassan Md, Marufhossain M, Bailey J, Ramamohanarao K Improving k-nearest neighbour classification with distance functions based on receiver operating characteristics.

[83] Babiloni F, Bianchi L, Semeraro F Mahalanobis distance-based classifiers are able to recognize EEG patterns by using few EEG electrodes.

[84] Mahalanobis PC (1936). On the generalized distance in statistics. *Proceedings National Institute of Science, India*.

[85] Martens SMM, Leiva JM (2010). A generative model approach for decoding in the visual event-related potential-based brain-computer interface speller. *Journal of Neural Engineering*.

[86] van Gerven MAJ, de Lange FP, Heskes T (2010). Neural decoding with hierarchical generative models. *Neural Computation*.

[87] Brodersen KH, Schofield TM, Leff AP (2011). Generative embedding for model-based classification of fMRI data. *PLOS Computational Biology*.

[88] Kemere C, Shenoy KV, Meng TH (2004). Model-based neural decoding of reaching movements: a maximum likelihood approach. *IEEE Transactions on Biomedical Engineering*.

[89] HB M (2012). Sequential Bayesian Inference. *Data Fusion: Concepts and Ideas*.

[90] Wu S, Chen D, Niranjan M, Amari SI (2003). Sequential Bayesian decoding with a population of neurons. *Neural Computation*.

[91] Yoon JW, Roberts SJ, Dyson M, Gan JQ (2009). Adaptive classification for brain computer interface systems using sequential monte carlo sampling. *Neural Networks*.

[92] Wang Y, Paiva AR, Príncipe JC, Sanchez JC (2009). Sequential Monte Carlo point-process estimation of kinematics from neural spiking activity for brain-machine interfaces. *Neural Computation*.

[93] Sorrentino A, Parkkonen L, Pascarella A, Campi C, Piana M (2009). Dynamical MEG source modeling with multi-target bayesian filtering. *Human Brain Mapping*.

[94] Sanger TD (2007). Bayesian filtering of myoelectric signals. *Journal of Neurophysiology*.

[95] Nie D, Wang XW, Shi LC, Lu BL EEG-based emotion recognition during watching movies.

[96] Cutrell E, Tan D BCI for passive input in HCI.

[97] Gao JF, Yang Y, Lin P, Wang P, Zheng CX (2010). Automatic removal of eye-movement and blink artifacts from eeg signals. *Brain Topography*.

[98] Joyce CA, Gorodnitsky IF, Kutas M (2004). Automatic removal of eye movement and blink artifacts from EEG data using blind component separation. *Psychophysiology*.

[99] Romero S, Mañanas MA, Barbanoj MJ (2008). A comparative study of automatic techniques for ocular artifact reduction in spontaneous EEG signals based on clinical target variables: a simulation case. *Computers in Biology and Medicine*.

[100] Chi YM, Wang YT, Wang Y, Maier C, Jung TP, Cauwenberghs G (2012). Dry and noncontact EEG sensors for mobile brain-computer interfaces. *IEEE Trans Neural Syst Rehabil Eng*.

[101] Liao LD, Wang IJ, Chen SF, Chang JY, Lin CT (2011). Design, fabrication and experimental validation of a novel dry-contact sensor for measuring electroencephalography signals without skin preparation. *Sensors*.

[102] Chi YM, Jung TP, Cauwenberghs G (2010). Dry-contact and noncontact biopotential electrodes: methodological review. *IEEE Reviews in Biomedical Engineering*.

[103] Lin CT, Liao LD, Liu YH, Wang IJ, Lin BS, Chang JY (2011). Novel dry polymer foam electrodes for long-term EEG measurement. *IEEE Transactions on Biomedical Engineering*.

[104] Kemp AH, Gray MA, Eide P, Silberstein RB, Nathan PJ (2002). Steady-state visually evoked potential topography during processing of emotional valence in healthy subjects. *NeuroImage*.

[105] Pollatos O, Kirsch W, Schandry R (2005). On the relationship between interoceptive awareness, emotional experience, and brain processes. *Cognitive Brain Research*.

[106] Baumgartner T, Esslen M, Jäncke L (2006). From emotion perception to emotion experience: emotions evoked by pictures and classical music. *International Journal of Psychophysiology*.

[107] Li M, Lu BL (2009). Emotion classification based on gamma-band EEG. *Conference Proceedings: IEEE Engineering in Medicine and Biology Society*.

[108] Lithari C, Frantzidis CA, Papadelis C (2010). Are females more responsive to emotional stimuli? A neurophysiological study across arousal and valence dimensions. *Brain Topography*.

[109] Park KS, Choi H, Lee KJ, Lee JY, An KO, Kim EJ (2011). Emotion recognition based on the asymmetric left and right activation. *International Journal of Medicine and Medical Sciences*.

[110] Degabriele R, Lagopoulos J, Malhi G (2011). Neural correlates of emotional face processing in bipolar disorder: an event-related potential study. *Journal of Affective Disorders*.

[111] Lin YP, Wang CH, Jung TP (2010). EEG-based emotion recognition in music listening. *IEEE Transactions on Biomedical Engineering*.

[112] Hosseini SA, Khalilzadeh MA, Naghibi-Sistani MB, Niazmand V Higher order spectra analysis of EEG signals in emotional stress states.

[113] Wang XW, Nie D, Lu BL (2011). EEG-based emotion recognition using frequency domain features and support vector machines. *Neural Information Processing*.

[114] Stelios K, Hadjidimitriou LJ (2012). Toward an EEG-based recognition of music liking using time-frequency analysis. *IEEE Transactions on Biomedical Engineering*.

